# Assessing the durability of a cash transfer on physical intimate partner violence and sexual relationships among adolescent girls and young women in rural South Africa

**DOI:** 10.1016/j.socscimed.2024.116948

**Published:** 2024-05-06

**Authors:** Allison K. Groves, Luwam T. Gebrekristos, Marie C.D. Stoner, F Xavier Gómez-Olivé, Kathleen Kahn, Audrey E. Pettifor

**Affiliations:** a Department of Community Health and Prevention, Drexel University, Philadelphia, PA, USA; b Department of Epidemiology and Biostatistics, Drexel University, Philadelphia, PA, USA; c Women’s Global Health Imperative, RTI International, Berkeley, CA, USA; d MRC/Wits Rural Public Health and Health Transitions Research Unit (Agincourt), School of Public Health, Faculty of Health Sciences, University of the Witwatersrand, Johannesburg, Gauteng, South Africa; e INDEPTH Network, Accra, Ghana; f University of North Carolina at Chapel Hill, Chapel Hill, NC, USA

## Abstract

**Introduction:**

Cash transfers can reduce adolescent girls and young women’s (AGYW) risk of intimate partner violence (IPV). In our own cash transfer intervention (HPTN 068), AGYW who received a cash transfer were less likely to experience IPV than non-recipients, in part because the cash reduced their engagement in sexual partnerships. This mixed-methods study builds on earlier findings to examine whether the protective effects were sustained after the cash ended and when the cash transfer was the most impactful.

**Methods:**

HPTN 068 was an experimental HIV prevention intervention trial. AGYW who participated completed 3 annual surveys during the intervention and an additional survey 2.5 years post-intervention. We used log-binomial regression models to assess the durability of the cash transfer on outcomes and included an interaction term in models to examine when effects were largest. We analyzed qualitative interviews conducted after the cash ended to contextualize findings.

**Results:**

Post-intervention, the relative risk of physical IPV was lower among AGYW who received it compared to those who did not, but not statistically significant (RR: 0.83, 95% CI: 0.62, 1.10). AGYW who received the cash transfer also had a lower relative risk of ever having had sex and of having any sexual partner in the last 12 months (RR: 0.94, 95% CI: 0.88, 1.01; RR: 0.94; 95% CI: 0.88, 0.99, respectively). The protective effect of the cash transfer on physical IPV was highest in Years 1 and 2 (RR: 0.64; 95% CI: 0.55–0.75 and RR: 0.65; 95% CI: 0.55–0.77, respectively). Qualitative data corroborated the quantitative findings.

**Conclusion:**

The cash transfer reduced AGYW’s risk of IPV, though effects were attenuated after the cash ended. Provision of cash during adolescence – a period when AGYW are highly susceptible to IPV and HIV – may empower them in their current relationship and yield long term health benefits.

## 1. Introduction

Cash transfer programs, or the provision of regular payments to individuals or households independent of employment, have played an important role in HIV prevention for women and adolescent girls in Sub Saharan Africa, both of whom are significantly burdened by HIV and poverty. Cash transfer programs to women and adolescent girls can decrease poverty-related stressors, shift economic resource allocation, and positively impact bandwidth and decision-making (Haushofer and Fehr, 2014; Mani et al., 2013). In turn, cash transfer programs can theoretically foster safer sexual partnerships and health-promoting behaviors, by increasing women and adolescent girls’ agency to negotiate condom use or decreasing their engagement in transactional sex (i.e., money for material needs/goods). Indeed, a recent meta-analysis of population-level cash transfer programs in countries with generalized HIV epidemics found that such programs yielded several positive effects for women, including decreased risk of sexually transmitted infections (STIs) and increased likelihood of recent HIV testing (Richterman and Thirumurthy, 2022). Cash transfer programs have also yielded positive effects for adolescent girls and young women (AGYW) between the ages of 15–24. A separate systematic review (Stoner et al., 2021) identified several cash transfer programs that reduced AGYW’s STI risk (Baird et al., 2010; Goodman et al., 2014) and delayed their sexual debut (Goodman et al., 2014; Abdoulayi et al., 2016; American Institutes for Research (AIR), 2015; Department of Social Development et al., 2018; Handa et al., 2014; Heinrich and Brill, 2015; Siaplay, 2012), though the effects of cash transfers on HIV biomarkers was mixed (Baird et al., 2010; Gorgens et al., 2020; Minnis et al., 2019; Pettifor et al., 2016a).

There is also evidence that cash transfer programs reduce women and adolescent girls’ risk of intimate partner violence (IPV), which is highly prevalent in Sub-Saharan Africa (Sardinha et al., 2022; Decker et al., 2014) and associated with increased risk of HIV acquisition (Jewkes et al., 2010; Li et al., 2014). Cash transfer programs have been theorized to reduce risk of IPV by decreasing household conflict, increasing economic security, and increasing women’s empowerment (Buller et al., 2018). While most of the evidence on cash transfers and women’s experience of IPV occurs in settings outside of Sub-Saharan Africa (Bobonis et al., 2013; Hidrobo et al., 2016; Hidrobo and Fernald, 2013), a Kenyan study that examined the impact of an unconditional cash transfer on IPV outcomes among adult women found that receipt of the cash transfer increased women’s empowerment (assessed using a composite measure, which included violence exposure) (Haushofer and Shapiro, 2016). Furthermore, in our own cash transfer study in rural South Africa (the HIV Prevention Trials Network (HPTN) study, HPTN 068), adolescent girls and their households each received a monthly cash transfer that was conditional on 80% school attendance, and receipt of the cash transfer reduced adolescent girls’ risk of IPV by 34% (Pettifor et al., 2016a).

The growing literature on cash transfer programs and HIV risk among women and adolescent girls, while important, suffers from three limitations. First, we have a limited understanding of the durability of cash transfer programs on HIV risk in general, and IPV in particular (i.e., are the protective effects of cash transfers programs sustained after the cash payments have ended?) To our knowledge, there is only one study that has examined the durability of a cash transfer on adult women’s risk of IPV in Bangladesh (Roy et al., 2019) and only one study that has examined the durability of a cash transfer on other sexual risk outcomes (but not IPV) among adolescent girls in Africa (Baird et al., 2019). Second and relatedly, there are gaps in understanding the durability of cash transfers for reducing both IPV and sexual risk, even though both occur within partnerships and have similar root causes of gender and economic inequities (Jewkes et al., 2010; Jewkes, 2010). And finally, there are no mixed methods or qualitative papers that examine adolescent girls’ perspectives on how cash transfers impact their sexual relationships, including their exposure to IPV, after the cash ends. Understanding the long-term benefits of cash transfers on IPV and sexual risk for AGYW in countries highly burdened by HIV can inform policies regarding when cash transfers should be delivered to maximize their impact.

Therefore, the purpose of this paper is to conduct a mixed-methods secondary data analysis to (1) examine the durability of a conditional cash transfer on South African AGYW’s exposure to physical IPV, sexual debut, and number of sexual partnerships two and a half years after the end of the cash transfer; (2) examine when the impacts of the cash transfer on outcomes were the greatest; and (3) explore AGYW’s perspectives on how the end of the cash transfer impacted their sexual partnerships, including their experiences of IPV within these partnerships.

## 2. Methods

### 2.1. Study design and sample

Data for the analyses come from the HIV Prevention Trials Network (HPTN) 068 study, a phase III randomized trial to determine whether cash transfers, conditional on school attendance, reduced AGYW’s risk of acquiring HIV (Pettifor et al., 2016b). AGYW aged 13–20 years who were in high school, not married, not pregnant, and residing in Agincourt Health and Socio-Demographic Surveillance Systems (AHDSS) catchment area in Mpumalanga Province in South Africa were enrolled in HPTN 068 (n = 2,533). For the current analysis, participants with prevalent HIV infection at baseline were excluded, yielding an analytic sample of 2,448 participants. Enrolled participants were consented and then randomized (1:1) into one of two arms: 1) received a monthly cash transfer conditional on attending at least 80% of school days in the past month, or 2) did not receive a monthly cash transfer. Young women in the intervention group received 100 rands (about $10 USD in 2012), and their parent/guardian received 200 rands (about $20) per month. The amount was purposively chosen to be on par with the income from the Child Support Grant (an existing state-sponsored unconditional cash transfer designed to ameliorate child poverty). Moreover, given that the average monthly per capita household expenditure at baseline was 295 Rand, the cash transfer represented a significant proportion of household consumption (Kilburn et al., 2019). During the intervention period, participants were followed until they graduated or until the end of the trial, whichever came first. Written informed consent for study participation was obtained from both young women (>18 years) and their parent or guardian. Written assent was obtained for minor female participants (<18 years). Institutional Review Board approval for this study was obtained from the University of North Carolina at Chapel Hill and the University of the Witwatersrand Human Research Ethics Committee as well as the Provincial Department of Health’s Research Ethics Committee.

### 2.2. Quantitative data collection

All participants completed HIV and HSV-2 testing and Audio Computer-Assisted Self-Interview (ACASI) surveys at the baseline visit, follow-up visits (annually for up to three years) and post-intervention visit (two and a half years after the cash transfer ended).

### 2.3. Measures

#### Outcomes.

Physical IPV in the last 12 months was measured using six items from the WHO modified conflict tactics scale (World Health Organization, 2005). Participants who reported at least one act of violence were coded as having experienced physical IPV. Participants who reported no violence across all six items were coded as not having experienced physical IPV. Sexual debut in the last 12 months, having a sexual partner in the last 12 months and having more than one sexual partner in the last 12 months were each measured separately using a single item.

#### Covariates.

Individual characteristics at baseline: age, experienced physical IPV in the last 12 months (yes versus no), ever experienced physical IPV (yes versus no), sexual debut (ever had sex versus never had sex), had a sexual partner in the last 12 months (yes versus no), had more than one sexual partner in the last 12 months (yes versus no), had a previous pregnancy (yes versus no), orphanhood (mother or father died versus neither died), had alcohol more than once a month (yes versus no). Household characteristics at baseline: food insecurity in the last 12 months (yes versus no) and being in the highest quartile for per capita household expenditure (yes versus no).

### 2.4. Statistical analysis

The post-intervention visit had a retention rate of 77.4%. Several baseline characteristics (study arm, alcohol use and household expenditures) were associated with attrition (see Supplementary materials). To account for attrition bias, we used an inverse propensity weighting technique and used these baseline characteristics to calculate attrition weights for the durability analyses. Propensity scores were calculated using logistic models with an indicator for retention as the outcome and identified baseline characteristics as predictors. A participant’s propensity score is her predicted probability of remaining in the study given the baseline characteristics and study arm.

We then calculated stabilized inverse propensity weights (IPW) using propensity scores. We calculated unstabilized weights by taking the inverse of the predicted probabilities. Unstabilized weights can get very large since it is the reciprocal of the predicted probabilities. Therefore, we created and used stabilized weights by multiplying unstabilized weights by the retention rate or 77.4%.

We assessed the durability of the intervention effects on the outcomes (measured at post-intervention visit) using log-binomial regression with stabilized weights, controlling for baseline age and time between baseline and post-intervention visit. To examine the effect of the cash transfer across all 4 study waves while accounting for repeated observations, we used log-binomial GEE models. To explore the effect of the cash transfer over time, we included an interaction term between study wave and study arm.

### 2.5. Qualitative data collection

In addition to completing surveys, 22 participants from the intervention arm were invited to complete an in-depth interview at the post-intervention visit. We purposively sampled girls who reported having one or more sexual partnerships during or after the cash transfer because we wanted to learn from their perspectives how receipt of the cash transfer impacted their sexual relationships over time, including after the intervention ended. All interviews were conducted by trained local research staff in native xiTsonga (the local language). Interviews were recorded, transcribed, and translated. Interviewers used a semi-structured guide to ask participants about each of the relationships they had during and after the cash transfer. Researchers used probes to understand IPV and sexual behavior within the relationships as well as participants’ perceptions on how the cash transfer impacted their engagement in these relationships.

### 2.6. Qualitative data analysis

After reading the transcripts, we first analyzed the data using a connecting technique (Maxwell and Miller, 2008) by generating a narrative summary of each participants’ relationship trajectory. These trajectories included a general timeline for each relationship and the dynamics of each relationship (including IPV and other HIV risk), with attention to both the timing of each relationship relative to the cash transfer and how receipt of the cash transfer influenced the relationship. Second, we used a categorizing technique (Maxwell and Miller, 2008; Miles and Huberman, 1994) by creating a matrix in which we summarized each participant’s relationship trajectory in a separate row and then compared data across participants. Through this process, we identified three patterns: (1) adolescent girls who experienced no IPV in their relationships over the entire study period, (2) adolescent girls who experienced decreased IPV within/across their relationships over the study period, and (3) adolescent girls who experienced ongoing or increased IPV within/across their relationships over the study period.

To integrate the qualitative and quantitative data and to understand whether and how these patterns of IPV were influenced by receipt of the cash transfer, we wrote interpretive memos [ (Birks et al., 2008)]. Throughout the analysis process, our interpretation was informed by our team’s extensive experience with HIV prevention research and practice in South Africa.

## 3. Results

### 3.1. Quantitative data findings

The median age of adolescent girls at baseline was 15 years old (IQR: 14–17) (Table 1). At baseline, nearly one in five adolescent girls had experienced physical IPV in their lifetime (17%) and one in ten had experienced physical IPV in the last 12 months (11%). At baseline, approximately one in four adolescent girls reported having had a sexual partner in the last 12 months (27%), though few adolescent girls reported having more than one sexual partner in the last 12 months (6%). Moreover, at the post-intervention visit, the weighted prevalence of HIV and HSV-2 were 8.9% and 23.0%, respectively.

Table 2 illustrates the weighted, bivariate and multivariable analyses reflecting the durable effects of the cash transfer on IPV and other sexual behaviors at the post-intervention visit two and a half years after the cash transfer had ended. While nearly one in ten AGYW reported any physical IPV in the last 12 months at the post-intervention visit, the prevalence and relative risk of physical IPV was lower among AGYW who received the cash transfer compared to those in the control arm, but not statistically significant (8% versus 10%; RR: 0.83, 95% CI: 0.62, 1.10). Nearly two-thirds of AGYW reported ever having had sex by the post-intervention visit and AGYW who received the cash transfer had a significantly lower prevalence and marginally lower relative risk of ever having had sex than AGYW in the control arm (63% versus 67%; RR: 0.94, 95% CI: 0.88, 1.01). Relatedly, the prevalence and relative risk of having any sexual partner in the last 12 months was also lower among AGYW who received the cash transfer than AGYW in the control arm (62% versus 66%; RR: 0.94; 95% CI: 0.88, 0.99). Finally, fewer than one in five AGYW had more than one sexual partner in the last 12 months, and AGYW who received the cash transfer were marginally less likely to report having multiple partners than AGYW in the control arm (13% vs 16%; RR: 0.82, 95% CI: 0.66, 1.02).

Table 3 shows the pooled effects of the cash transfer on outcomes across all time points. Receipt of the cash transfer had a protective effect on AGYW’s risk of physical IPV (RR: 0.70; 95% CI: 0.62, 0.78) across the entire study period. Receipt of the cash transfer also had a protective, but marginally significant effect on AGYW’s sexual debut (RR: 0.94; 95% CI: 0.88, 1.01), likelihood of having a sexual partner (RR: 0.94; 95% CI: 0.88, 1.00), and likelihood of having more than one sexual partner (RR: 0.84; 95% CI: 0.70, 1.01) across all time points.

Finally, Fig. 1 shows the effect of the cash transfer on physical IPV over the study period by study arm. Across both study arms, IPV decreased over time. The effect of the cash transfer on physical IPV was the highest in Year 1 and Year 2 (Fig. 1, RR: 0.64; 95% CI: 0.55–0.75 and RR: 0.65; 95% CI: 0.55–0.77, respectively). Compared to Year 1, the effect of the cash transfer was reduced by 22% in Year 3 and was marginally significant (RR: 0.78; 95% CI: 0.59, 1.02). The post-intervention wave had the smallest effect with a reduction of 16% from Year 1 and was not statistically significant (RR: 0.84; 95% CI: 0.63–1.11).

### 3.2. Qualitative findings

Qualitative data corroborated the quantitative findings by revealing that young women were most likely to describe the ways that receiving the cash transfer reduced their risk of exposure to IPV while they were actively receiving cash. A majority of young women experienced no violence while receiving the cash transfer, and many described how the cash transfer positively impacted their relationship dynamics. The cash also provided independence and autonomy for some young women who were in violent relationships during the intervention. For example, one young woman described how receiving cash enabled her to negotiate condom use with one of her sexual partners,

“I told my boyfriend that we must use protection and he refused maybe he will push me to have sex with him because he knew that he was giving me money. You see something like that he will oppress me because of his money but since I was having my own money. It made me say f– off you can stay with your money. I have my own money.”

When her partner was not amenable to using condoms, this participant ended the relationship. She started dating someone else – a *“respected man*” – who did not abuse her. Another young woman described how she stopped dating multiple men at the same time (one of whom was violent) while receiving the cash transfer.

Most young women continued to feel empowered in their sexual relationships after the end of the cash transfer. Some who had experienced violence from their partners and ended the relationship while receiving the cash transfer described that they were now in healthy sexual partnerships and were not experiencing violence in these new partnerships. Others who had experienced violence from their partners and ended the relationship while receiving the cash transfer purposefully abstained from sexual partnerships once the cash transfer was over.

For a few young women, the end of the cash transfer negatively affected their financial independence. One young woman described relying on her partner for cash post-intervention, *“I was worried after the cash has stopped because I was buying whatever I want with this Swa Koteka money ….if I want to buy something now, I have to save the money that I get from my boyfriend.”* Similarly, another young woman who was not in a sexual partnership after the end of the intervention was contemplating initiating a new relationship to increase her access to cash, *“at this moment it pushes me to have a boyfriend because there is no use, the money [at home] is little.”* Finally, and relatedly, one individual who was in a violent relationship after the cash transfer ended felt unable to leave her partner because she was financially reliant on him.

## 4. Discussion

The cash transfer reduced AGYWs’ risk of IPV, and the effect of the cash transfer was the strongest while AGYW were receiving cash. There was some evidence that the cash transfer continued to protect AGYW from IPV even after the cash ended, particularly given that IPV decreased over time for all AGYW. Furthermore, the cash transfer had durable effects on sexual partnerships such that even after the cash transfer ended, AGYW who had received the cash transfer were less likely to have a recent sexual partner than AGYW who did not. Findings were corroborated by young women’s descriptions of their experiences of IPV and their sexual relationships during and after receipt of the cash transfer. Specifically, young women described how receiving cash enabled them to engage in healthy relationships and in some cases, to leave violent relationships. While young women described how they continued to feel empowered in their selection of sexual partners even after the cash ended, a few felt reliant on partners for cash post-intervention, which may have affected their ongoing exposure to violence. These findings – that the protective effects of cash transfers on IPV is strongest while cash is being provided – add to a growing body of evidence of the importance of ongoing poverty mitigation for adolescent health. The potential of social protection programs (like cash transfers) on IPV and sexual partnerships may be especially relevant during high-risk periods, such as when AGYW are finishing secondary school and transitioning to adulthood.

In the original study, we found that AGYW who had received the cash transfer had greater power over whether and how they engaged in sexual relationships (Kilburn et al., 2018). In the current study, we found that some of these effects persisted two and a half years after the cash transfer ended. That is, AGYW who received the cash transfer were less likely than those who did not receive the cash transfer to report having a sexual partner after the cash transfer ended. The AGYW who received the cash transfer were also somewhat less likely than their peers to report multiple sexual partners or sexual debut after the cash transfer ended. To our knowledge there are no other cash studies that have looked at long-term impacts on engagement in sexual partnerships, though a study of the long-term effects of an unconditional cash transfer in Malawi reported no long-term differences in HIV prevalence (or child marriage) after the unconditional cash transfer ceased (Baird et al., 2019).

The protective effects of the cash transfer on IPV weakened after the cash transfer program concluded. These findings may be because the small monthly cash transfer that adolescent girls received (i.e., ~$10 USD) did not lead to long-term wealth. In a rural context like Agincourt, which is characterized by high unemployment for youth (Wilkinson et al., 2017), many adolescent girls described a lack of economic independence after the cash transfer ended and, thus, relied once again on their boyfriends for cash post-intervention. These findings are consistent with one other examination of the durability of cash transfers on IPV among adult women. In that study, Bangladeshi women who received cash transfers had similar levels of physical violence six to ten months after the program ended as compared to women who did not receive the cash transfer (Roy et al., 2019). Alternatively, it is possible that the decrease in IPV across all AGYW over time weakened our ability to detect a significant difference between CCT recipients and non-recipients. To our knowledge, there are no other evaluations of the durability of cash transfers for adolescent girls’ risk of IPV. Future research on whether cash transfers for AGYW have durable effects on IPV in other contexts – or among subpopulations of AGYW (e.g., those who are food insecure) – will enable us to better understand how cash transfers might optimize AGYWs’ sexual relationships (and reduce their susceptibility to IPV), not only while cash transfers are in place, but also over time.

Furthermore, several studies have reported durable impacts of cash transfers (or other subsidies) on women and adolescent girls’ IPV and HIV outcomes when they are combined with other programs or services. For example, women in Bangladesh who received the cash transfer alongside a behavior change communication program experienced sustained reductions in IPV not only six to ten months after the program ended, but four years later as well (Roy et al., 2022). Additionally, adolescent girls in Kenya who received an educational subsidy program combined with an HIV curriculum experienced fewer Herpes Simplex Virus (HSV-2) infections seven years later than those who did not receive the combination intervention, and HSV-2 is a known risk factor for HIV (Duflo et al., 2015). To our knowledge, no interventions have looked at the durability of cash transfers alongside IPV-specific services, such as screening and referral, and/or safety planning. Future research should examine whether such combination interventions have durable effects on IPV for AGYW.

Our finding that a modestly sized cash transfer reduced AGYW’s risk of IPV (even though the effects waned over time) expands the limited evidence on structural interventions to reduce IPV. The effects of this transformative poverty approach are especially promising given that adolescence constitutes an important window of opportunity for IPV prevention: it is often the first time that AGYW experience violence in sexual relationships, which affects their relationships into early adulthood (Cui et al., 2013). A recent systematic review of interventions to reduce IPV among adolescents identified five other interventions in sub-Saharan Africa which had at least one positive effect on adolescent girls’ risk of violence (McNaughton Reyes et al., 2021), of which only one included an economic empowerment component (i.e., the program included vocational training alongside life skills training sessions) (Bandiera et al., 2012). The other effective violence prevention interventions were resource intensive in different ways than cash transfers (i.e., they included between 5 and 21 skill building sessions with youth and often included other intervention components as well). Future research should consider the cost effectiveness of different approaches to reduce IPV among adolescent girls and young women as well as optimal strategies to implement these programs.

This study has several limitations. First, IPV and sexual behavior are both highly sensitive outcomes and may be misreported (Palen et al., 2008; Cullen, 2020). Second, all participants were in school at study enrollment; therefore, our findings may not be generalizable to adolescent girls who are not enrolled in school. A third limitation is that we did not qualitatively sample participants who were not in a sexual partnership (or who had never been in a sexual partnership) after the intervention ended. Therefore, we do not understand how those young women who received the cash transfer opted out of sexual partnerships after the cash ended and whether opting out was a strategy they used to decrease their exposure to violence (and HIV).

## 5. Conclusions

A growing body of literature indicates the potential of cash transfers to reduce gender inequities in HIV and IPV in Sub-Saharan Africa. However, few studies have examined the durability of cash transfers on HIV and IPV, particularly among AGYW in high-burden HIV settings like South Africa [ (Gibbs et al., 2017)]. Our study broadens understanding of when cash matters most for adolescent relationships by highlighting (1) that cash transfers programs provide AGYW the strongest protection from IPV while they are receiving the cash, and (2) that receiving a cash transfer has positive and durable impacts on engaging in sexual partnerships even after the cash has ended. In response to these findings and given the ongoing challenges with reducing HIV and IPV among AGYW in the region, policymakers might consider implementing longer lasting cash transfer programs specifically for girls during their adolescence. Further research is needed to determine the optimal length of cash transfer programs for adolescent girls, their cost effectiveness relative to other IPV and HIV reduction strategies, and whether other supports could bolster the protective effects of cash for AGYW after the cash ends.

## Supplementary Material

Appendix A. Supplementary data

## Figures and Tables

**>Fig. 1.**
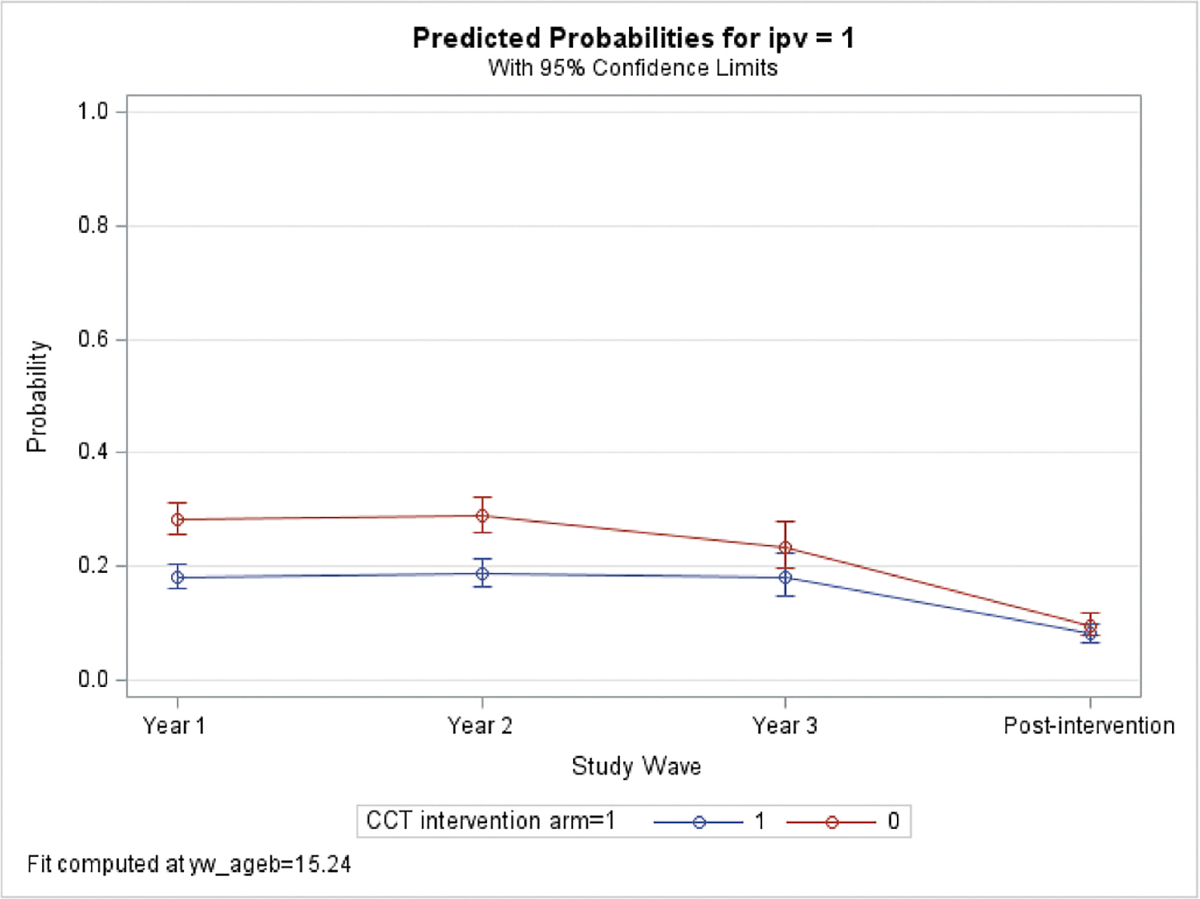
Predicted probabilities of IPV.

**Table 1:** Baseline characteristics by study arm.

	Total N = 2,448	Treatment Arm N = 1,225	Control Arm N = 1,223

Baseline Characteristics	N (%) or Mean (SD) or Median (IQR)		
Age	15 (14–17)	15 (14–17)	15 (14–16)
Ever physical IPV	415 (17%)	219 (18%)	196 (16%)
Any physical IPV in the last 12 months	254 (11%)	136 (11%)	118 (10%)
Ever had sex	649 (27%)	324 (27%)	325 (27%)
Had a sexual partner in the last 12 months	645 (27%)	316 (26%)	329 (27%)
More than 1 sexual partner in the last 12 months	145 (6%)	72 (6%)	73 (6%)
Ever pregnant	206 (9%)	105 (9%)	101 (8%)
Had alcohol more than once in the last month	55 (2%)	31 (3%)	24 (2%)
Experience food insecurity	829 (34%)	399 (33%)	430 (36%)
Highest quartile for household expenditure	611 (25%)	296 (24%)	315 (26%)
Orphaned	486 (20%)	251 (21%)	235 (19%)

**Table 2:** Weighted Prevalence and effects of CCT on sexual behaviors at post-intervention visit.

	Total N = 1,964	Treatment Arm N = 1,022	Control Arm N = 941	p-value	RR (95% CI)^a^	p-value
		
	N (%)					

Any physical IPV in the last 12 months	173 (9%)	79 (8%)	95 (10%)	0.19	0.83 (0.62, 1.10)	0.19
Sexual debut	1221 (65%)	590 (63%)	632 (67%)	0.04	0.94 (0.88, 1.01)	0.07
Had a sexual partner in the last 12 months	1203 (64%)	580 (62%)	625 (66%)	0.03	0.94 (0.88, 0.99)	0.04
More than 1 sexual partner in the last 12 months	275 (15%)	123 (13%)	152 (16%)	0.07	0.82 (0.66, 1.02)	0.08

a Weighted log-binomial regression controlling for age and time since baseline.

**Table 3:** Pooled effects of the CCT on sexual behaviors and relationship characteristics.

	Pooled	
	
	RR (95% CI)^a^	p-value

Any physical IPV in the last 12 months	0.70 (0.62, 0.78)	<0.0001
Sexual debut	0.94 (0.88, 1.01)	0.08
Had a sexual partner in the last 12 months	0.94 (0.88, 1.00)	0.06
More than 1 sexual partner in the last 12 months	0.84 (0.70, 1.01)	0.06

a GEE log-binomial regression controlling for age.

## Data Availability

The authors do not have permission to share data.

## Appendix A. Supplementary data

Supplementary data to this article can be found online at https://doi.org/10.1016/j.socscimed.2024.116948.
