# Supplementary material for: Assessing the durability of a cash transfer on physical intimate partner violence and sexual relationships among adolescent girls and young women in rural South Africa
Source: Soc Sci Med. Author manuscript; Available in PMC 2026 Feb 23. (PMC12927584; doi:10.1016/j.socscimed.2024.116948)
Supplement: Appendix A. Supplementary data [file NIHMS2140621-supplement-Appendix_A__Supplementary_data.docx]

**Table 1 Bivariate Associations between baseline characteristics and study dropout**

|  | Dropout 22.6%; N=554 | No Dropout 77.4%; N=1,894 | p-value |
| --- | --- | --- | --- |
| Study Arm |  |  |  |
| Treatment | 19.4% (238) | 80.6% (987) | **0.0002** |
| Control | 25.8% (316) | 74.2% (907) |  |
| Baseline Characteristics |  | |  |
| Age | 15.5 (1.7) | 15.5 (1.6) | 0.73 |
| Ever physical IPV |  |  |  |
| Yes | 24.1% (100) | 75.9% (315) | 0.36 |
| No | 22.0% (436) | 78.0% (1544) |  |
| Any physical IPV in the last 12 months |  |  |  |
| Yes | 24.4% (62) | 75.6% (192) | 0.41 |
| No | 22.2% (474) | 77.9% (1666) |  |
| Ever had sex |  |  |  |
| Yes | 23.4% (152) | 76.6% (497) | 0.56 |
| No | 22.3% (399) | 77.7% (1390) |  |
| Had a sexual partner in the last 12 months |  |  |  |
| Yes | 23.4% (151) | 76.6% (494) | 0.49 |
| No | 22.1% (390) | 77.9% (1377) |  |
| More than 1 sexual partner in the last 12 months |  |  |  |
| Yes | 20.7% (30) | 79.3% (115) | 0.60 |
| No | 22.5% (511) | 77.5% (1756) |  |
| Ever pregnant |  |  |  |
| Yes | 27.7% (57) | 72.3% (149) | 0.07 |
| No | 22.1% (489) | 77.9% (1724) |  |
| Had alcohol more than once in the last month |  |  |  |
| Yes | 36.4% (20) | 63.6% (35) | **0.02** |
| No | 22.2% (530) | 77.8% (1856) |  |
| Experience food insecurity |  |  |  |
| Yes | 20.8% (172) | 79.3% (657) | 0.14 |
| No | 23.4% (374) | 76.6% (1224) |  |
| Highest quartile for household expenditure |  |  |  |
| Yes | 26.2% (160) | 73.8% (451) | **0.01** |
| No | 21.4% (393) | 78.6% (1441) |  |
| Orphaned |  |  |  |
| Yes | 24.3% (118) | 75.7% (368) | 0.28 |
| No | 22.0% (427) | 78.0% (1515) |  |
